# Altered oxidative stress and carbohydrate metabolism in canine mammary tumors

**DOI:** 10.14202/vetworld.2016.1489-1492

**Published:** 2016-12-31

**Authors:** K. Jayasri, K. Padmaja, M. Saibaba

**Affiliations:** 1 Department of Veterinary Biochemistry, College of Veterinary Science, Tirupati, Andhra Pradesh, India; 2 Department of Surgery and Radiology, College of Veterinary Science, Tirupati, Andhra Pradesh, India

**Keywords:** canine mammary tumor, fructose-1, 6-bisphosphatase, glucose-6-phosphatase, glucose-6-phosphate dehydrogenase, hexokinase, thiobarbituric reactive substances

## Abstract

**Aim:**

Mammary tumors are the most prevalent type of neoplasms in canines. Even though cancer induced metabolic alterations are well established, the clinical data describing the metabolic profiles of animal tumors is not available. Hence, our present investigation was carried out with the aim of studying changes in carbohydrate metabolism along with the level of oxidative stress in canine mammary tumors.

**Materials and Methods:**

Fresh mammary tumor tissues along with the adjacent healthy tissues were collected from the college surgical ward. The levels of thiobarbituric acid reactive substances (TBARS), glutathione, protein, hexose, hexokinase, glucose-6-phosphatase, fructose-1, 6-bisphosphatase, and glucose-6-phosphate dehydrogenase (G6PD) were analyzed in all the tissues. The results were analyzed statistically.

**Results:**

More than two-fold increase in TBARS and three-fold increase in glutathione levels were observed in neoplastic tissues. Hexokinase activity and hexose concentration (175%) was found to be increased, whereas glucose-6-phosphatase (33%), fructose-1, 6-bisphosphatase (42%), and G6PD (5 fold) activities were reduced in tumor mass compared to control.

**Conclusion:**

Finally, it was revealed that lipid peroxidation was increased with differentially altered carbohydrate metabolism in canine mammary tumors.

## Introduction

Mammary tumors are the most common and prevalent type of neoplasms in canine species. The etiology of mammary tumor is multifactorial. Continuous exposure to xeno-estrogens present in water, food, and air has been reported to favor their bioaccumulation in the mammary tissue [1]. Mammalian epithelial cells activate xeno-estrogens to highly toxic reactive oxygen species (ROS) which are directly or indirectly involved in the process of mutagenesis [1]. Although the ROS production is counteracted by antioxidant systems of the body, excess production of free radicals are found to be involved in both initiation and promotion of multistage carcinogenesis [2]. ROS can cause DNA damage, activate procarcinogens, and alter the cellular antioxidant defense system [2].

Increase in the concentration of ROS contributes to oncogenic mutations which in turn alter metabolism and induce aerobic glycolysis [3]. The metabolic alterations in the tumor cell which happen under unique tumor microenvironment are directed toward the increased synthesis of biomolecules, high energy supply, and tightened maintenance of cellular redox status [4]. The biochemical reprogramming in cancer cell derives glucose utilization for energy function by upregulation of glycolytic enzymes [5,6]. Increased oxidative stress/ROS results in shunting of glucose away from glycolysis toward pentose phosphate pathway (PPP) [7]. PPP is considered important for tumor genesis as it provides reduced nicotinamide adenine dinucleotide phosphate (NADPH) for macromolecule biosynthesis and ROS detoxification as well as ribose-5-phosphate for nucleic acid synthesis [8]. Glucose-6-phosphate dehydrogenase (G6PD), a key metabolic enzyme, participating in PPP, is tightly associated with development and progression of a variety of tumors [9].

It is anticipated that recognizing metabolic enzymes critical for tumor cell proliferation and survival, will help in identifying novel therapeutic targets. The key metabolic alterations involved in canine mammary tumors have not been reported earlier. Hence, this study was conducted to understand the alterations in oxidative stress and to identify the key metabolic alterations of carbohydrate metabolism in canine mammary tumor tissues.

## Materials and Methods

### Ethical approval

The approval from the institutional animal ethics committee was not required since the tissues excised during surgery were used for analysis.

### Sample collection and analysis

Six fresh tumor tissues were collected along with the surrounding healthy tissues from the college surgical ward and stored at −40°C until further analysis. The affected dogs were in 7-9 years age group. The history revealed ovariohysterectomy in half of the cases. The dogs were completely free from any other pathology. The tumor tissues were histologically graded as adenocarcinoma Grade III. The tissues were homogenized (10%) in 1.15% KCl and analyzed for thiobarbituric acid reactive substances (TBARS) [10]. Similarly, 10% homogenate of tissues was prepared in 0.2 M sodium phosphate buffer pH 8.0 for the estimation of glutathione [11]. Tissue homogenate was prepared in 0.1 M Tris-HCl buffer pH 7.0, and the cytosolic fraction was collected by centrifuging at 10,000 rpm for 30 min. The concentration of hexose, protein and activities of hexokinase, glucose-6-phosphatase, fructose-1, 6-bisphosphatase, G6PD was estimated in the cytosol of all the tissues [12-17].

### Statistical analysis

The data were analyzed statistically using two-tailed t-test [18] using statistical software SPSS.

## Results

The levels of TBARS were found to be elevated (two-fold increase) in the mammary tumor tissues compared to healthy tissue indicating a higher degree of lipid peroxidation (Table-1). The levels of the tissue antioxidant, reduced glutathione (GSH) were significantly increased (p<0.001) in mammary tumor tissues compared to normal tissues (Table-1). The hexose content of tumor mass was found to be higher (175%) compared to that of normal tissue. The activity of hexokinase, an enzyme of glycolysis was enhanced (50%) in tumor mass compared to control tissue (Table-1). The activities of glucose-6-phosphatase and fructose-1, 6-bisphosphatase (enzymes of gluconeogenesis) were significantly low (p<0.001) in tumor tissue compared to normal tissues (Table-1). The activity of glucose-6- phosphate dehydrogenase was found to be significantly reduced (p<0.001) in tumor mass compared to the normal healthy tissue.

**Table-1:** Altered carbohydrate metabolism in canine mammary tumors.

Parameter	Normal mammary gland	Mammary tumor
TBARS (µmoles/g tissue)	13.73^a^±1.37	49.17^b^±3.81
GSH (mg/100 g tissue)	32.0^a^±1.34	134^b^±9.6
Hexose (mg/g tissue)	184^a^±15.54	506.67^b^±28.0
Hexokinase (µ moles of glucose phosphorylated/min/mg protein)	0.41^a^±0.02	0.66^b^±0.03
Glucose-6-phosphatase (µ moles of inorganic phosphorus released/min/mg protein)	0.067^a^±0.0041	0.045^b^±0.0013
Fructose-1, 6-bisphosphatase (µ moles of inorganic phosphorus released/min/mg protein	7.04^a^±0.15	3.96^b^±0.15
G6PD (mIU/mg protein)	0.245^a^±0.011	0.054^b^±0.002

Values are mean±standard error.

a,b Means are significantly different (p<0.001). G6PD=Glucose-6-phosphate dehydrogenase, TBARS=Thiobarbituric reactive substances, GSH=Reduced glutathione

## Discussion

Increase in lipid peroxidation of canine mammary tumors observed in this study can be attributed to overproduction of oxygen free radicals. It is similar to the report of increased TBARS levels in the neoplastic tissues of dogs affected with mammary tumors [19]. Serum MDA was reported to be higher in dogs with mammary tumors compared to healthy dogs [20]. Contradictory results were also reported with no significant difference in serum TBARS levels in dogs with and without malignant mammary tumors [21-23]. Chronic increases in ROS may trigger transformation and contribute to cancer progression by amplifying genomic instability. In a normal cell, proliferation resulting from growth factor stimulation requires ROS signaling. It has been reported that neoplastic transformation is associated with an increase in the basal level of ROS-mediated signaling which may result in constitutive activation of such cell proliferation pathway [24]. Further, excessive production of oxygen free radicals due to increased metabolism may also contribute to elevated TBARS [1].

Glutathione, a thiol-containing compound plays a major role in the detoxification of reactive intermediates [4]. Increased lipid peroxidation in our study is accompanied by enhanced glutathione levels. Szczubial *et al*. [22] reported no significant difference in serum thiol groups in dogs with and without mammary tumor. Increased GSH expression is reported in canine mammary tumors without ulceration, not metastatic tumors, and low mortality [25]. Intracellular GSH maintains the reduced status of thioredoxin which activates ribonucleotide reductase, a key enzyme essential for DNA synthesis [1]. Elevation of GSH levels is an early proliferative response that has been found to change the thiol redox status of the cell, resulting in activation of genes essential for G_1_ to S transition in cell cycle [1]. Hence elevated GSH in canine mammary tissues may be believed to contribute both to antioxidant defense and cell proliferation.

The best characterized metabolic phenotype observed in the tumor cells is the Warburg effect, a shift from ATP generation through oxidative phosphorylation to ATP generation through glycolysis even under normal oxygen concentrations [26]. Although the ATP production by glycolysis is more rapid, it is far less efficient in terms of ATP generated per unit glucose consumed. Hence, this shift demands a high rate of glucose uptake to meet energy and biosynthetic needs [27]. The increased hexose content in the tumor tissues compared to normal tissues in our study indicates increased glucose uptake by the cancer cells. The increase in 2-fluoro-2-deoxy glucose (FDG) uptake by mammary tumors of genetically engineered mice was reported to be dictated by initiating oncogenes [28]. The high glucose metabolism of cancer cells is reported to be caused by a combination of hypoxia-responsive transcription factors, activation of oncogenic proteins, and the loss of tumor suppressor function. It was reported that oncogenic transformation of cultured mammalian cells causes a rapid increase of glucose transport [29].

Significant increase in the activity of hexokinase in the tumor tissues compared to normal tissues indicates a rapid rate of glycolysis in canine mammary tumor tissues. Similar findings were reported in breast tumor cases [30]. A significant correlation has been reported between increased FDG uptake and enhanced HKII expression in mammary tumors induced by different oncogenic pathways in mice [28]. Bryson *et al*. [31] have reported that glucokinase and hexokinase II have the ability to suppress apoptosis through the interaction with mitochondria and suppression of cytochrome C release. Increase in hexokinase activity in our study may explain its role both in glycolytic flux and in cancer cell survival.

Jagadeesan *et al*. [32] also observed decreased levels of glucose-6-phosphatase and F-1, 6-bisphosphatase in the liver of patients with breast cancer. Similar results were observed in our study in canine mammary tumor tissues compared to normal tissue. These two are the enzymes involved in gluconeogenesis producing glucose from noncarbohydrate sources. The decreased activity of these two enzymes may be due to shunting of glycolytic intermediates into the subsidiary pathways to fuel the biosynthetic pathways [33]. The activity of G-6-P dehydrogenase was decreased in the cancer tissues compared to healthy tissue. It is an important enzyme in oxidative phase of hexose monophosphate pathway and a source of NADPH which is essential for both reductive biosynthesis and biosynthesis of glutathione. Our results are contrary to Wang *et al*. [9] who reported an elevation in G6PD activity in esophageal squamous cell carcinoma compared to normal tissue and its expression was found to be associated with poor prognosis. This could be due to the alternative pathways operating to supply reducing equivalents in canine mammary tumors. Glutaminolysis might be involved in the provision of NADPH for fatty acid, nucleotide synthesis and for maintenance of glutathione pool [4]. Alternative pathways operating in canine mammary tumors might be responsible for maintenance of elevated GSH pool even with decreased G-6-phosphate dehydrogenase activity in canine mammary tumors.

## Conclusion

We conclude that the high basal level of oxidative stress with altered carbohydrate metabolism was observed in canine mammary tumor. G6PD independent supply of reducing quivalents is envisaged in canine mammary tumors. However, much more clinical data are required involving the other enzymes of carbohydrate metabolism to discern definite enzyme pattern.

## Authors’ Contributions

KJ, KP, MS conceived the study, collected the samples, carried out the laboratory work, analyzed the data, drafted and revised the manuscript. All authors have read and approved the final manuscript.
